# Antioxidant Cascade Nanoenzyme Antagonize Inflammatory Pain by Modulating MAPK/p‐65 Signaling Pathway

**DOI:** 10.1002/advs.202206934

**Published:** 2023-02-17

**Authors:** Yuejuan Ling, Dekang Nie, Yue Huang, Mengyuan Deng, Qianqian Liu, Jinlong Shi, Siguang Ouyang, Yu Yang, Song Deng, Zhichao Lu, Junling Yang, Yi Wang, Rongqin Huang, Wei Shi

**Affiliations:** ^1^ Department of Neurosurgery Research Center of Clinical Medicine Neuro‐Microscopy and Minimally Invasive Translational Medicine Innovation Center Affiliated Hospital of Nantong University Medical School of Nantong University Nantong University 226001 Nantong P. R. China; ^2^ Institute of Pain Medicine and Special Environmental Medicine Nantong University Nantong 226001 P. R. China; ^3^ Department of Neurosurgery Yancheng First Hospital Affiliated Hospital of Nanjing University Medical School The First people's Hospital of Yancheng 224001 Yancheng P. R. China; ^4^ Center for Advanced Low‐dimension Materials State Key Laboratory for Modification of Chemical Fibers and Polymer Materials College of Chemistry Chemical Engineering and Biotechnology Donghua University Shanghai 201620 P. R. China; ^5^ Department of Pharmaceutics School of Pharmacy Key Laboratory of Smart Drug Delivery Ministry of Education Fudan University Shanghai 215537 P. R. China

**Keywords:** biomimetic nanoenzyme system, inflammatory pain, neuroinflammation, reactive oxygen species scavenging

## Abstract

Chronic pain has attracted wide interest because it is a major obstacle affecting the quality of life. Consequently, safe, efficient, and low‐addictive drugs are highly desirable. Nanoparticles (NPs) with robust anti‐oxidative stress and anti‐inflammatory properties possess therapeutic possibilities for inflammatory pain. Herein, a bioactive zeolitic imidazolate framework (ZIF)‐8‐capped superoxide dismutase (SOD) and Fe_3_O_4_ NPs (SOD&Fe_3_O_4_@ZIF‐8, SFZ) is developed to achieve enhanced catalytic, antioxidative activities, and inflammatory environment selectivity, ultimately improving analgesic efficacy. SFZ NPs reduce tert‐butyl hydroperoxide (t‐BOOH)‐induced reactive oxygen species (ROS) overproduction, thereby depressing the oxidative stress and inhibiting the lipopolysaccharide (LPS)‐induced inflammatory response in microglia. After intrathecal injection, SFZ NPs efficiently accumulate at the lumbar enlargement of the spinal cord and significantly relieve complete Freund's adjuvant (CFA)‐induced inflammatory pain in mice. Moreover, the detailed mechanism of inflammatory pain therapy via SFZ NPs is further studied, where SFZ NPs inhibit the activation of the mitogen‐activated protein kinase (MAPK)/p‐65 signaling pathway, leading to reductions in phosphorylated protein levels (p‐65, p‐ERK, p‐JNK, and p‐p38) and inflammatory factors (tumor necrosis factor [TNF]‐α, interleukin [IL]‐6, and IL‐1*β*), thereby preventing microglia and astrocyte activation for acesodyne. This study provides a new cascade nanoenzyme for antioxidant treatments and explores its potential applications as non‐opioid analgesics.

## Graphical abstract

SOD&Fe_3_O_4_@ZIF‐8 (SFZ) nanoparticles (NPs), are obtained by encapsulating superoxide dismutase (SOD) and Fe_3_O_4_ NPs in zeolitic imidazolate framework (ZIF)‐8 NPs. SFZ NPs via intrathecal injection are degraded in a weakly acidic environment. Then, oxidative stress, inflammatory cytokines and the mitogen‐activated protein kinase/p‐65 signaling pathway is blocked, ultimately preventing the glial cell activation and relieving the inflammatory pain.

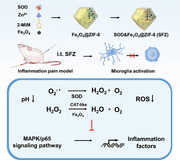

## 1 Introduction

Neuropathic pain, especially inflammatory neuralgia, severely affects the quality of life.^[^ ^1^ ^]^ It is commonly characterized by allodynia and anaphylaxis and is considered the most difficult pain to treat. In the inflammatory environment, the center of nociceptive sensory neurons releases many peroxides (reactive oxygen and nitrogen free radicals), inducing a strong oxidative stress response.^[^ ^2^ ^]^ Additionally, spinal glial cells are activated in abundance, producing reactive oxygen species (ROS), inflammatory factors, and other neurotoxic substances that act on the corresponding receptors or ion channels to activate multiple signaling pathways,^[^ ^3^ ^]^ leading to increased neuronal sensitivity and excitability, which aggravate pain. Presently, anti‐inflammatory drugs and opioid analgesics are often used in clinical treatment; however, these drugs cause hormonal level disturbances, gastrointestinal discomfort, addiction, and other side effects.^[^ ^4^ ^]^ Notably, nano‐targeted drug delivery systems have very good prospects in various diseases; however, studies of their application in chronic pain are few.^[^ ^5^ ^]^ There is an urgent need to develop drugs with strong antioxidant and anti‐inflammatory abilities and ideal physicochemical properties to treat inflammatory pain.

Superoxide dismutase (SOD),^[^ ^6^ ^]^ as the main internal antioxidant enzyme in the body, can catalyze the superoxide anion free radicals into hydrogen peroxide (H_2_O_2_) and oxygen (O_2_), reduce the level of cellular oxidative stress, and play the role of an antioxidant and anti‐inflammatory agent.^[^ ^7^ ^]^ However, during neuroinflammation, endogenous antioxidants' levels are insufficient to eliminate excess ROS, resulting in the inability to eliminate oxidative stress in spinal glial cells. Therefore, the exogenous supplementation of SOD to remove excessive ROS and relieve chronic pain is a promising strategy.^[^ ^8^ ^]^ Nevertheless, this strategy faces two dilemmas: 1) in vivo stable delivery of SOD molecules with resolved enzyme activity should be addressed; 2) synergistic enzyme catalysis should be designed to convert the produced H_2_O_2_ catalyzed by SOD enzyme into lower oxidation O_2_ for maximal ROS elimination.^[^ ^9^ ^]^ Recently, increasing evidence has shown that inorganic antioxidant enzyme nanoparticles (NPs) can treat multiple diseases and show better stability and efficacy than natural enzymes.^[^ ^10^ ^]^ Furthermore, Fe_3_O_4_ NPs—the most widely used nanoenzyme—have SOD‐ and catalase (CAT)‐like activities that convert superoxide to H_2_O_2_ and eventually to O_2_.^[^ ^11^ ^]^ Additionally, local Fe_3_O_4_ NPs administration might relieve inflammatory pain in mice by attenuating inflammatory cell infiltration and pro‐inflammatory signaling and scavenging microenvironment free radicals.^[^ ^12^ ^]^ However, Fe_3_O_4_ NPs is a preliminary exploration in analgesia, whose performances and mechanisms required further improvement and exploitation.

Zeolite imidazole acid frame‐8 (ZIF‐8) is a representative metal‐organic framework material assembled by 2‐methylimidazole ligands and zinc ions, which is non‐toxic and biocompatible.^[^ ^13^ ^]^ Importantly, ZIF‐8 NPs could encapsulate a variety of biological macromolecules, such as proteins (enzymes) and DNA, through a simple, low‐cost biomimetic mineralization process preventing their leakage and maintaining their biological activity.^[^ ^14^ ^]^ Notably, ZIF‐8 NPs decompose easily under acidic conditions (pH 5–6), which is conducive to efficiently delivering and releasing composite materials in a weakly acidic inflammatory environment.^[^ ^15^ ^]^ All these suggest that ZIF‐8 NPs are an excellent candidate for constructing multienzyme catalytic platforms; however, it has seldom been exploited for synergistic pain relief applications.^[^ ^16^ ^]^

**Scheme 1:**
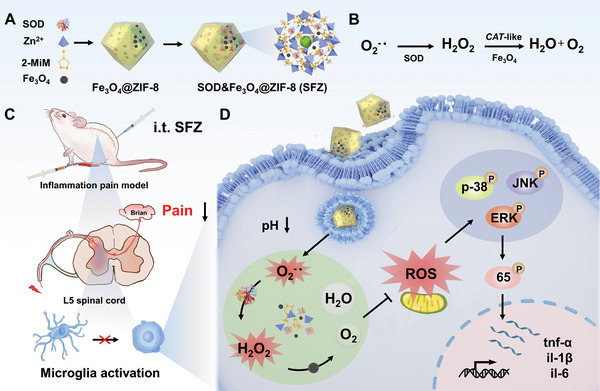
Schematic illustration of the synthetic route of SOD&Fe_3_O_4_@ZIF‐8 NPs with multi‐enzyme mimicking activities and its analgesic mechanism of anti‐oxidative and anti‐inflammatory effects on inflammatory pain.

Therefore, we explored a simple co‐assembly strategy to simultaneously encapsulate SOD and Fe_3_O_4_ NPs in ZIF‐8 NPs, obtaining an antioxidant cascade nanoenzyme, SOD&Fe_3_O_4_@ZIF‐8 (SFZ) (shown in **Scheme** **1**). This nanoenzyme could reduce undesired ROS release from inflammation‐activated glial cells and decrease the inflammatory factors for acesodyne via intrathecal injection. Typically, during an inflammatory response in the spinal cord, SFZ NPs could be degraded in a weakly acidic inflammatory environment to release the loaded SOD and Fe_3_O_4_ NPs, which would then be endocytosed by inflammatory cells. After that, the ROS (O_2_^−•^) generated by the inflammatory cells is converted to H_2_O_2_ and O_2_ through the SOD and Fe_3_O_4_‐mediated cascade catalytic reactions, thereby reducing oxidative damage in glial cells and alleviating central sensitization. In this study, the release of inflammatory cytokines—tumor necrosis factor‐alpha (TNF‐*α*), interleukin (IL)‐6, and IL‐1*β*—was inhibited, and the mitogen‐activated protein kinase (MAPK)/p‐65 signaling pathway was blocked, preventing the glial cell activation and relieving the inflammatory pain. Overall, this antioxidant cascade nanoenzyme is a promising strategy for treating inflammatory pain and other diseases caused by oxidative stress.

## 2 Results and Discussion

### 2.1 Synthesis and Characterization of SFZ and Other Counterparts

**Figure 1:**
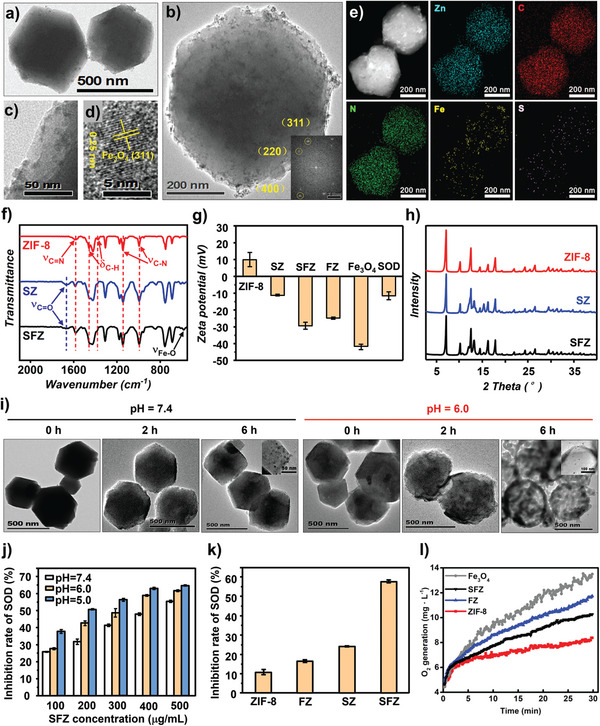
Structural characterization and pH‐responsive release of SFZ NPs. a,b) TEM images, c,d) HRTEM images, e) STEM images and EDX elements mappings of SFZ NPs. The inset in (b) is the ED pattern of SFZ NPs. f) FTIR spectra, g) zeta potentials (*n* = 3), and h) XRD patterns of different preparations. i) TEM images of SFZ NPs incubated in different pH solutions for varied time periods. j) SOD enzymatic activities (inhibition rates of SOD) of different SFZ NPs concentrations in different pH solutions detected by the SOD kit (*n* = 3). k) SOD enzymatic activities (Inhibition rates of SOD) of different nano‐preparations detected by the SOD kit (*n* = 3). l) Time curve of O_2_ generation from different nano‐preparations.

First, highly dispersed and small Fe_3_O_4_ NPs with distinct crystallinity was synthesized to prepare SFZ NPs (Figure S1, Supporting Information). Next, Fe_3_O_4_ NPs and SOD enzymes were incorporated into ZIF‐8 NPs via co‐assembly with Zn^2+^ and 2‐methylimidazole to obtain SFZ NPs. The resulting SFZ NPs exhibited a rhombic polyhedron‐like morphology with a particle size of 400–500 nm (**Figure** **1**a). Additionally, the well‐distributed dot‐like NPs with obvious lattice fringes (indexing as the (311) plane of Fe_3_O_4_) in the whole SFZ NPs indicates the uniform incorporation of Fe_3_O_4_ NPs (Figure 1b–d and Figure S2, Supporting Information); this was further revealed by the dispersed white dots in the scanning transmission electron microscopy (STEM) image, and the emerged Fe element in energy dispersive X‐ray (EDX) mappings (Figure 1e). Furthermore, the emerging S element that was uniformly spiked in the Zn—C—N matrix implies the simultaneous incorporation of SOD in SFZ NPs. To consolidate these results, we compared the Fourier transform infrared (FTIR) spectra for SFZ NPs (Figure 1f) to those of pure ZIF‐8 NPs and SOD‐incorporated ZIF‐8 (SZ NPs). The extra C=O stretching vibration and the emerged Fe—O stretching vibration confirm SOD and Fe_3_O_4_ NPs incorporation in SFZ NPs, respectively. Moreover, the variations in zeta potentials (Figure 1g) and sample colors (Figure S3, Supporting Information) in the different counterparts further indicate SOD and Fe_3_O_4_ NPs encapsulations. Notably, although the emerged S 2p (even P 2p) and Fe 2p signals in the X‐ray photoelectron spectra (XPS) revealed the presence of SOD and Fe_3_O_4_ NPs in SFZ NPs, respectively, these signals were relatively weak. This implies that most SOD and Fe_3_O_4_ NPs incorporations were in the inner part of SFZ NPs compared with other counterparts; therefore, they could not be detected using XPS surface analysis (Figure S4, Supporting Information). Despite the inner incorporation of SOD and Fe_3_O_4_ NPs in SFZ NPs, it did not deteriorate the ordered crystalline ZIF‐8 framework, confirmed by the nearly equal X‐ray diffraction (XRD) for all of these components (Figure 1h). Finally, ≈5.6 wt% SOD and 5.8 wt% Fe_3_O_4_ NPs were estimated in the SFZ NPs according to the comparative thermogravimetric analysis (TGA) results of different components (Figure S5, Supporting Information). These results suggest the efficient encapsulation of SOD and Fe_3_O_4_ NPs into ZIF‐8 NPs, which could serve as a robust vehicle to stably deliver the enzyme and NPs.

### 2.2 pH‐Responsive Release and Multienzymes Catalytic Activity of SFZ NPs In Vitro

Owing to the pH‐sensitive ZIF‐8 framework and SOD/Fe_3_O_4_ NPs incorporations, SFZ NPs gradually dissociated in solution at pH 6 to release the encapsulated SOD/Fe_3_O_4_ NPs (Figure 1i). After that, SFZ NPs exhibited a concentration‐ and pH‐dependent SOD enzyme activity as detected using the SOD kits, where the SFZ NPs had the maximal inhibition ratio among all of the counterparts, possibly due to the synergistic SOD enzyme‐like activity from Fe_3_O_4_ NPs besides that from the incorporated SOD (Figure 1j,k). Furthermore, Fe_3_O_4_ NPs catalyzed the O_2_ generation in the H_2_O_2_ solution owing to its CAT enzyme‐like activity (Figure 1l). After incorporating Fe_3_O_4_ NPs and SOD into ZIF‐8 NPs, the resulting NPs catalyzed the O_2_ generation, which was notably higher than that of pure ZIF‐8 NPs. The enzyme‐like activity of SFZ NPs was inferior to those of pure Fe_3_O_4_ NPs and SZ NPs, possibly due to the inadequate exposure of the incorporated active sites and SOD enzyme‐catalyzed H_2_O_2_ evolution without O_2_ generation, respectively.^[^ ^17^ ^]^ However, this reduced O_2_ generation and SOD enzyme‐like activity suggest the cascaded catalysis for ROS elimination (O_2_^−•^ and H_2_O_2_ removal) (Scheme 1b), indicating the potential benefits in relieving oxidative stress for inflammatory pain prevention.

### 2.3 Uptake and Disassembly of SFZ NPs in Cells

**Figure 2:**
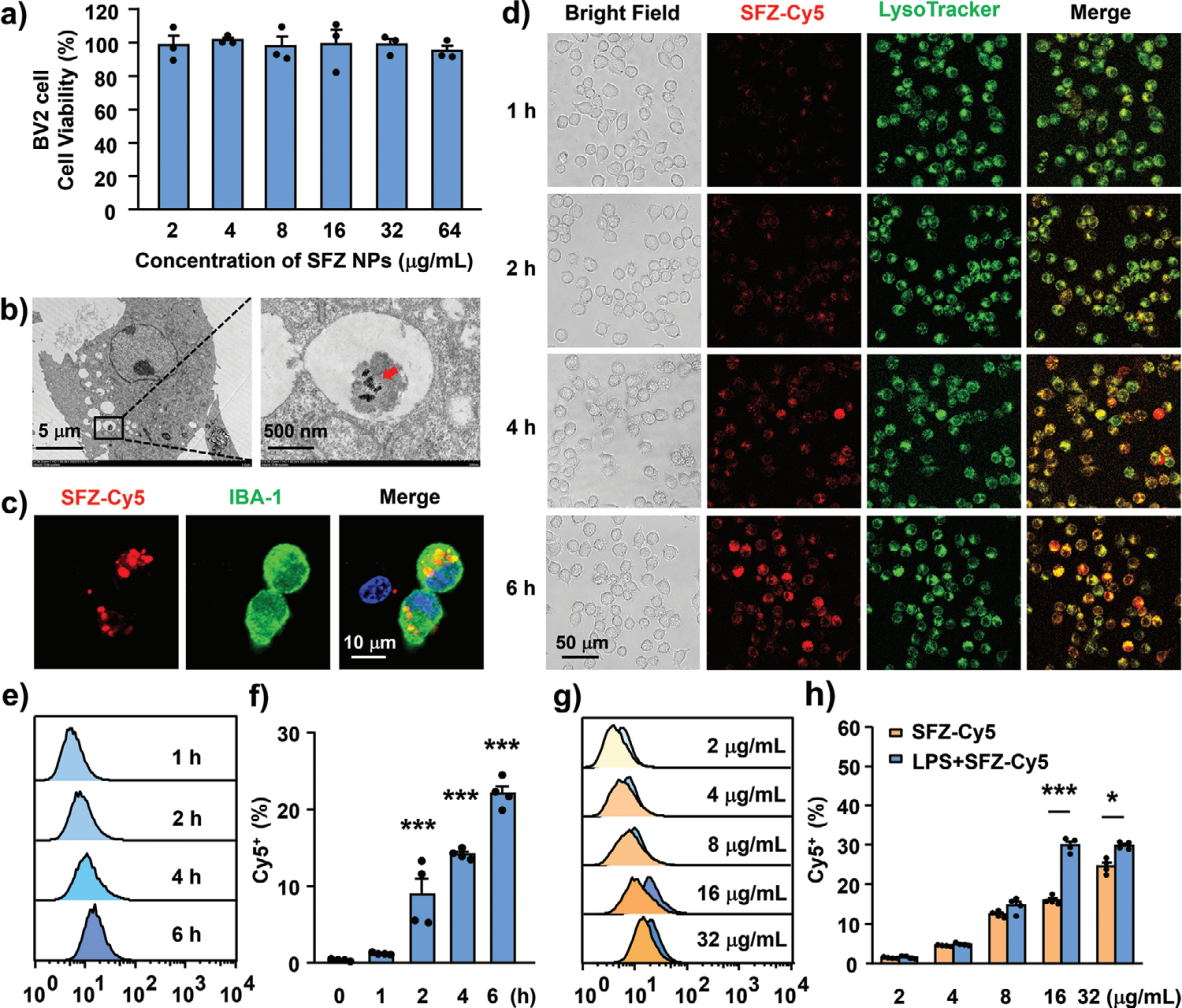
The cell viability and uptake of SFZ NPs in microglia cells. a) The cell viability of BV2 cells with SFZ NPs of different concentrations for 24 h (*n* = 3). b) TEM image of SFZ NPs internalized in BV2 cells. BV2 cells were incubated with SFZ NPs (16 µg mL^−1^) for 6 h. c) Localization of SFZ‐Cy5 NPs in IBA‐1 and DAPI after incubation with microglia cells for 6 h. d) Colocalization of SFZ‐Cy5 NPs and lysosome tracker in BV2 cells at different time points. e) Representative graph of cellular uptake intensity of SFZ‐Cy5 NPs with different time points in BV2 cells, f) quantitative analysis of Cy5^+^ by flow cytometry. ***p < 0.001, versus 1 h. One‐way ANOVA followed by Bonferroni's tests (*n* = 4). g) Representative graph of cellular uptake intensity of SFZ‐Cy5 NPs with different concentrations in naive and LPS‐induced BV2 cells, h) quantitative analysis of Cy5^+^ by flow cytometry. *p < 0.05, ***p < 0.001, LPS+SFZ‐Cy5 versus SFZ‐Cy5. Student's *t*‐test (*n* = 4).

The above results demonstrate that multi‐enzymatic NPs could be obtained through the proper encapsulation of SOD and Fe_3_O_4_ NPs in ZIF‐8 NPs. As innate immune cells of the central nervous system, microglia are extremely sensitive to changes in the inflammatory microenvironment and are rapidly activated in the spinal cord after chronic pain.^[^ ^18^ ^]^ Therefore, we investigated the performance of SFZ NPs in microglia. First, the cell counting kit (CCK‐8) assay was used to assess the viabilities of BV2 cells, primary microglia, and primary astrocytes cultured in different concentrations of SFZ NPs (**Figure** **2**a and Figure S6a,b, Supporting Information). The incubation of SFZ NPs had no obvious cytotoxicity against the three cell lines at <3.2 µg mL^−1^. Similarly, ZIF‐8 NPs and other related NPs had no obvious cytotoxicity against BV2 cells (Figure S6c, Supporting Information). These suggest the good biocompatibility of SFZ NPs. Next, we examined the uptake and localization of SFZ NPs in microglia using transmission electron microscopy (TEM) and fluorescence imaging. In TEM images (Figure 2b), SFZ NPs are found extracellularly and in the cytoplasm. Moreover, Cyanine 5 (Cy5)‐labeled SFZ NPs were localized in IBA‐1‐stained cytoplasm but not 4′,6‐diamidino‐2‐phenylindole (DAPI)‐stained nuclei (Figure 2c). These results indicate that part of the SFZ NPs was successfully endocytosed into microglia.

The investigation demonstrates that lysosome‐mediated endocytosis is an important mechanism for NPs to enter cells.^[^ ^19^ ^]^ To further investigate whether SFZ NPs were localized to lysosomes, we labeled the lysosomes of BV2 cells with a green fluorescent lysosomal probe. Furthermore, the fluorescence images show that Cy5‐labeled SFZ NPs (red fluorescence) started to enter lysosomes after 1 h incubation (Figure 2d). After that, the red fluorescence intensity gradually increased and co‐labeled with the green fluorescence of LysoTracker with a longer incubation time. Moreover, the flow cytometry results showed that with the incubation of SFZ‐Cy5 NPs for a longer time or at a higher concentration, the fluorescence intensity of intracellular Cy5 was higher. These suggest that the SFZ NPs could enter the cells in a time‐ and concentration‐dependent manner via lysosome‐mediated endocytosis (Figure 2e–h). Notably, studies have shown the significantly improved uptake of NPs by activated microglia, enhancing their therapeutic effect under inflammatory pathological conditions, consistent with our findings. Compared with normal culture conditions, when lipopolysaccharide (LPS) activated BV2 cells, the uptake capacity of SFZ‐Cy5 NPs was significantly increased (Figure 2g,h).

### 2.4 SFZ NPs Resist Intracellular Oxidative Stress through Cascade Catalytic Activity

**Figure 3:**
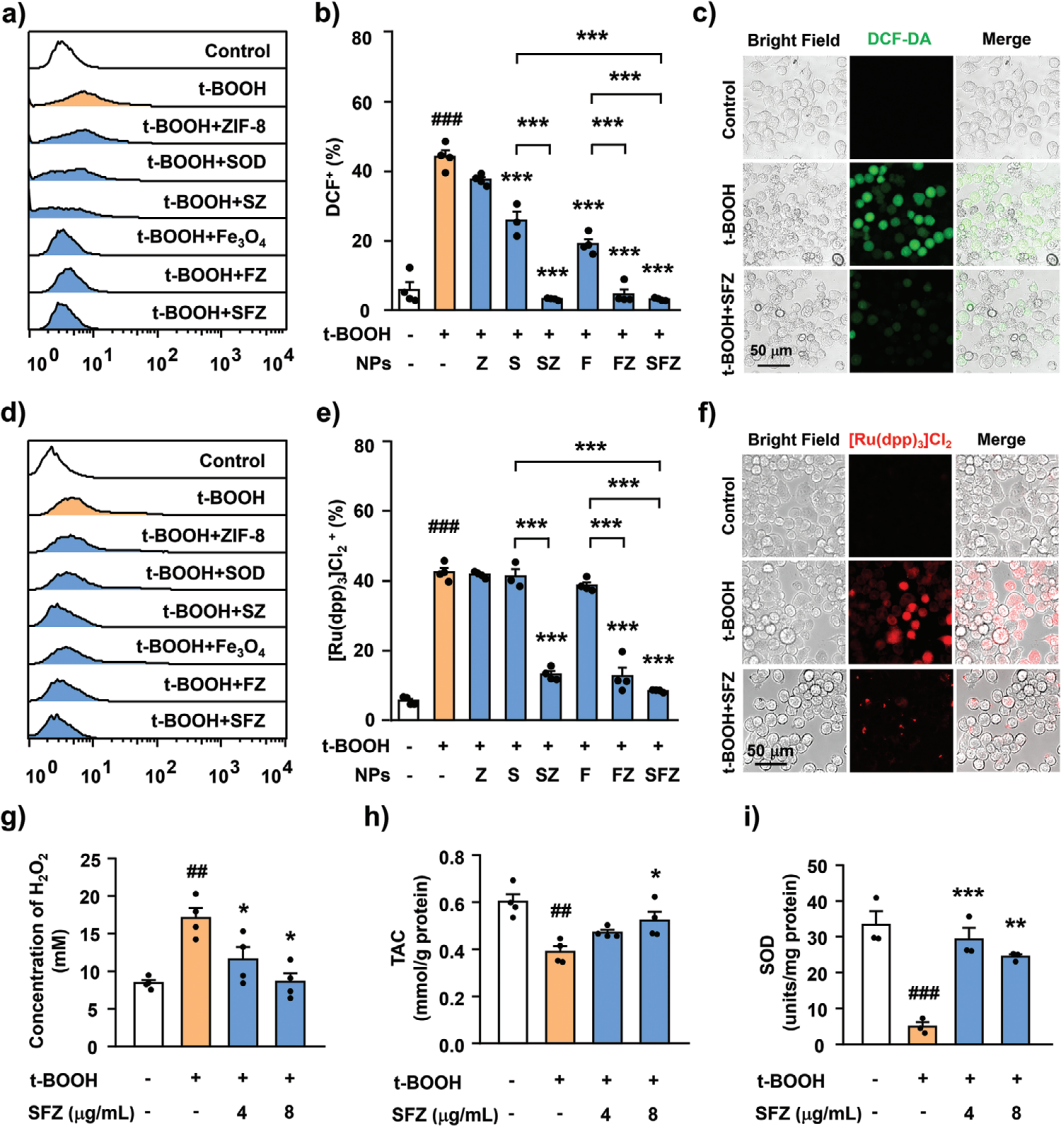
Effects of SFZ NPs on t‐BOOH‐induced oxidative stress in BV2 cells. a) Representative graph of different nanoparticles (ZIF‐8, SOD, SZ, Fe_3_O_4_, FZ, and SFZ NPs) to scavenge ROS induced by t‐BOOH in BV2 cells, b) quantitative analysis of DCF^+^ by flow cytometry. *^###^p* < 0.001, t‐BOOH versus PBS. ***p < 0.001, versus t‐BOOH, SZ versus SOD, SFZ versus SOD, FZ versus Fe_3_O_4_, SFZ versus Fe_3_O_4_. c) Confocal microscopy images showing relative ROS levels in BV2 cells by H2DCFDA probes. Scale bars, 50 µm. d) Representative graph of the oxygen‐generating capacity of different nanoparticles (ZIF‐8, SOD, SZ, Fe_3_O_4_, FZ, and SFZ NPs) in BV2 cells, e) quantitative analysis of Ru(dpp)_3_]Cl_2_^+^ by flow cytometry. *^###^p* < 0.001, t‐BOOH versus PBS. ***p < 0.001, versus t‐BOOH, SZ versus SOD, SFZ versus SOD, FZ versus Fe_3_O_4_, SFZ versus Fe_3_O_4_. One‐way ANOVA followed by Bonferroni's tests (*n* = 4). f) Confocal microscopy images showing relative O_2_ levels in BV2 cells by oxygen indicator probes. Scale bars, 50 µm. g–i) Levels of g) H_2_O_2_, h) TAC, and i) SOD in t‐BOOH‐induced BV2 cells treated with SFZ NPs at different concentrations. *^##^p* < 0.01, *^###^p* < 0.001, t‐BOOH versus PBS. *p < 0.05, **p < 0.01, ***p < 0.001, versus t‐BOOH, one‐way ANOVA, followed by Bonferroni's test (*n* = 4).

Oxidative stress caused by ROS is closely related to microglial activation, and the activated microglia can further produce ROS, forming a vicious circle.^[^ ^20^ ^]^ We investigated the role of SFZ NPs in preventing this vicious circle by evaluating the ROS and oxidative stress levels in microglia after SFZ NPs treatments using an H2DCFDA probe. tert‐butyl hydroperoxide (t‐BOOH) is a commonly used oxidant that can induce oxidative damage in cells.^[^ ^19^, ^21^ ^]^ Quantitative flow cytometry results showed that t‐BOOH could induce ROS generation in microglia in a concentration‐dependent manner (Figure S7a,b, Supporting Information). However, SFZ NPs treatment could scavenge the ROS, which positively correlated with the NP concentrations. At 16 µg mL^−1^ SFZ NPs, the t‐BOOH‐induced oxidative damage was almost completely reversed (Figure S7c,d, Supporting Information). Notably, the ROS scavenging capability of SFZ NPs was much higher than those of the other counterparts (SOD, Fe_3_O_4_ NPs, SZ NPs, and FZ NPs), implying the synergistic cascade catalytic effects (**Figure** **3**a,b). Additionally, confocal microscopy results showed that SFZ NPs inhibited t‐BOOH‐induced ROS generation in microglia (Figure 3c). These results demonstrate that SFZ NPs had high antioxidant activity.

The second step in ROS scavenging is CAT enzyme‐like activity, which catalyzes H_2_O_2_ to produce water and O_2_. To examine this performance of SFZ NPs, the intracellular O_2_ content was measured using the [Ru(dpp)_3_]Cl_2_ probe.^[^ ^22^ ^]^ Flow cytometry results showed that the degree of hypoxia in the microglia of the single t‐BOOH‐induced group was significantly higher than that of the control group. However, after pretreatments with SZ NPs, FZ NPs, and SFZ NPs, the hypoxia was reversed, indicated by the significantly reduced [Ru(dpp)_3_]Cl_2_ fluorescence in microglia (Figure 3d,e). Similarly, confocal microscopy results revealed that SFZ NPs increased t‐BOOH‐induced O_2_ production in microglia (Figure 3f) with CAT‐like activity. Particularly, compared with the SOD and Fe_3_O_4_ NPs groups, the SFZ NPs group had more enhanced O_2_ generation ability in microglia. This revealed that the cascade enzymatic catalytic activities stabilized via ZIF‐8 NPs entrapment.

ROS (superoxide anion and H_2_O_2_) can cause severe oxidative damage to cellular lipids, cell membranes, proteins, and DNA, as shown in many studies.^[^ ^23^ ^]^ Antioxidants scavenge these free radicals through enzymatic and non‐enzymatic mechanisms and prevent cell oxidative stress. We verified the antioxidant capacity of SFZ NPs as a nanozyme by measuring their antioxidant capacity using an H_2_O_2_ assay kit, a total antioxidant capacity assay kit, and a total SOD activity detection kit. Consistent with the above results, SFZ NPs significantly inhibited t‐BOOH‐induced overproduction of H_2_O_2_ (Figure 3g). Additionally, the total antioxidant capacity (Figure 3h) and SOD enzyme‐like activity (Figure 3i) were also significantly enhanced after SFZ NPs incubation as compared with the t‐BOOH group. Consequently, SFZ NPs inhibited ROS production in microglia and alleviated oxidative stress.

### 2.5 SFZ NPs Alleviate the Inflammatory Environment of Microglia by Inhibiting the Activation of the MAPK Signaling Pathway and the Expression of Inflammatory Factors

The above findings prompted us to further explore the intracellular mechanism behind the remarkable cascade catalytic activity of SFZ NPs. Various articles have documented that the MAPK signaling pathway is one of the molecular mechanisms involved in neuroinflammation, including extracellular response kinases (ERKs), c‐Jun N‐terminal kinases (JNKs), and p38‐MAPKs.^[^ ^24^ ^]^ Moreover, ROS and the inflammatory environment play an important role in this process.^[^ ^25^ ^]^ First, ROS activates PLC‐*γ* and Src, directly and indirectly, phosphorylates Ras and Raf, leading to ERK activation. Furthermore, activated ERK indirectly modulates ROS levels by inducing p22phox, resulting in increased ROS production. Second, ROS activates ASK1 through multiple mechanisms, leading to the activation of JNK and p38.^[^ ^26^ ^]^ Notably, p38 and JNK kinases are primarily expressed in the microglia and astrocytes, respectively. Additionally, excessive ROS generated during oxidative metabolism can trigger and aggravate the inflammatory process including the activation of nuclear factor kappa B (NF‐*κ*B) and the production of pro‐inflammatory factors.^[^ ^27^ ^]^ Therefore, we speculated that SFZ NPs alleviated oxidative stress by interfering with the MAPK signaling pathway and inhibiting pro‐inflammatory factor production.

**Figure 4:**
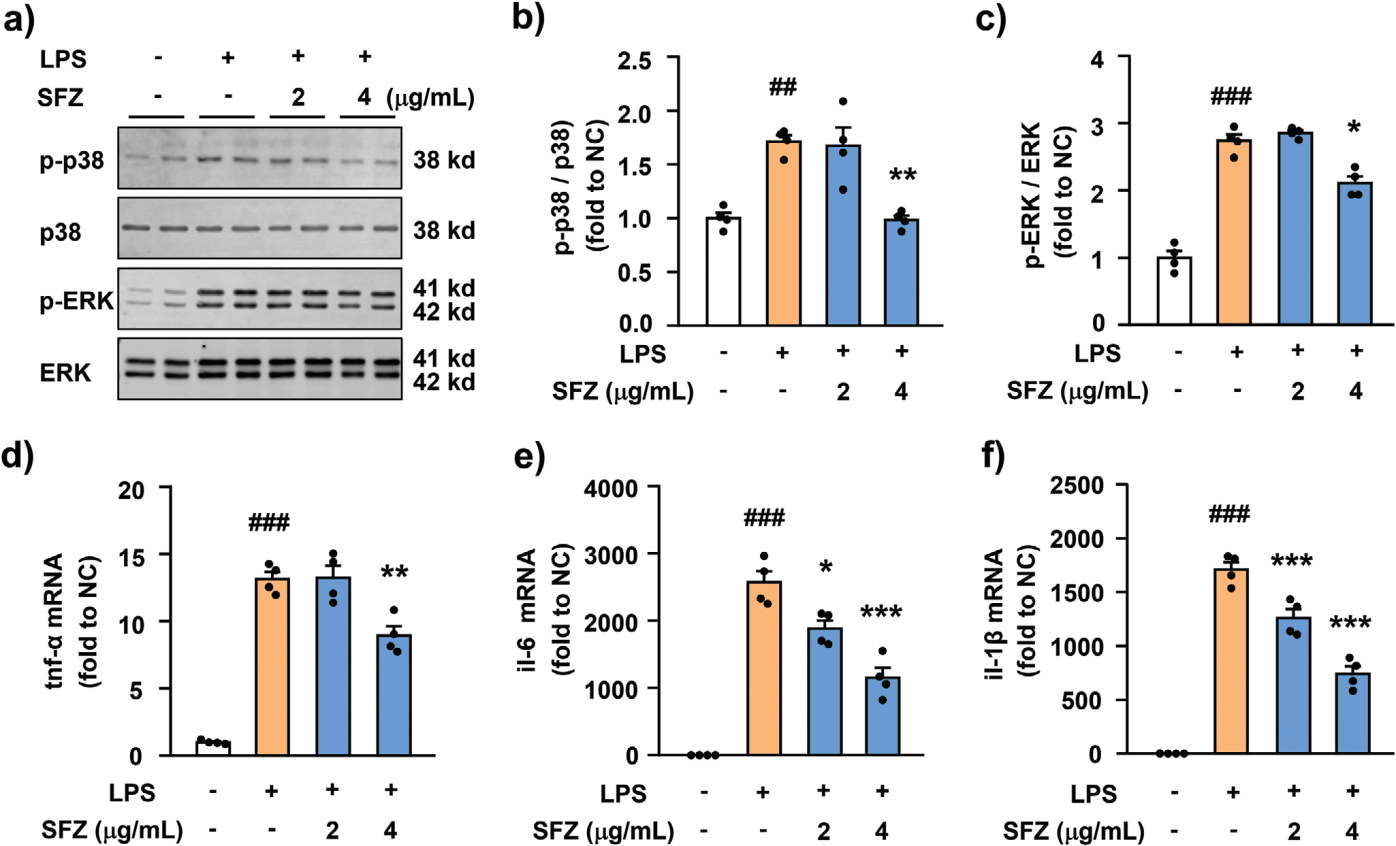
Effects of SFZ NPs on neuroinflammation in primary microglia cells. a–c) Expression and protein quantification of a,b) p‐p38 and a,c) p‐ERK in primary microglial cells with different treatments characterized by western blotting. *^###^p* < 0.001, t‐BOOH versus PBS. ***p < 0.001, versus t‐BOOH, one‐way ANOVA, followed by Bonferroni's test (*n* = 4). d–f) The mRNA levels of d) TNF‐*α*, e) IL‐6, and f) IL‐1*β* in primary microglial cells with different treatments. *^###^p* < 0.001, t‐BOOH versus PBS. *p < 0.05, **p < 0.01, ***p < 0.001, versus t‐BOOH, one‐way ANOVA, followed by Bonferroni's test (*n* = 4).

To verify the above conjecture, a western blot was used to detect the expression levels of phosphorylated ERK (p‐ERK) and p38 in primary microglia. **Figure** **4**a–c shows that LPS significantly induced increased levels of p‐ERK and p‐p38 compared with the control groups; however, their expressions were significantly downregulated after 4 µg mL^−1^ SFZ NPs treatment. Similarly, the LPS‐induced p‐ERK and p‐JNK expressions were significantly downregulated by SFZ NPs treatment in primary astrocytes (Figure S8a–c, Supporting Information). These results indicate that SFZ NPs inhibited the activation of the MAPK signaling pathway. Furthermore, the downstream pro‐inflammatory factor mRNA expression was examined by quantitative PCR. Figure 4d–f and Figure S8d–f, Supporting Information show that 2 and 4 µg mL^−1^ SFZ NPs could significantly downregulate the LPS‐induced upregulated inflammatory factors in microglia and astrocytes and 4 µg mL^−1^ SFZ NPs was more effective than 2 µg mL^−1^. As mentioned above, we revealed the underlying molecular mechanism by which SFZ NPs suppress glial oxidative stress. As an outstanding multi‐enzymatic activity of NPs, SFZ NPs played a key role in the antioxidant and anti‐inflammatory conditions in the glial cells.

### 2.6 Intrathecal Injection of SFZ NPs was Mainly Distributed on the Inflammatory Side of the Spinal Cord

To explore the therapeutic performance of SFZ NPs in vivo, we induced inflammatory pain in mice by plantar injection of complete Freund's adjuvant (CFA). Several studies have shown that CFA‐induced inflammatory pain models have significant nociceptive and inflammatory response characteristics, often used to screen new compounds for anti‐inflammatory pain activity.^[^ ^28^ ^]^ After inducing inflammatory pain in mice using CFA, we injected SFZ NPs intrathecally and continuously monitored mechanical nociceptive hypersensitivity to evaluate its effect in treating inflammatory pain. Notably, intrathecal injection into the mouse L5 intervertebral space facilitates the direct delivery of NPs to the spinal glia. Clinically, this administration can be performed with a lumbar puncture. Since the drug enters the subarachnoid space directly without passing through the blood–brain barrier, the concentration in the cerebrospinal fluid is high, producing a good effect.

**Figure 5:**
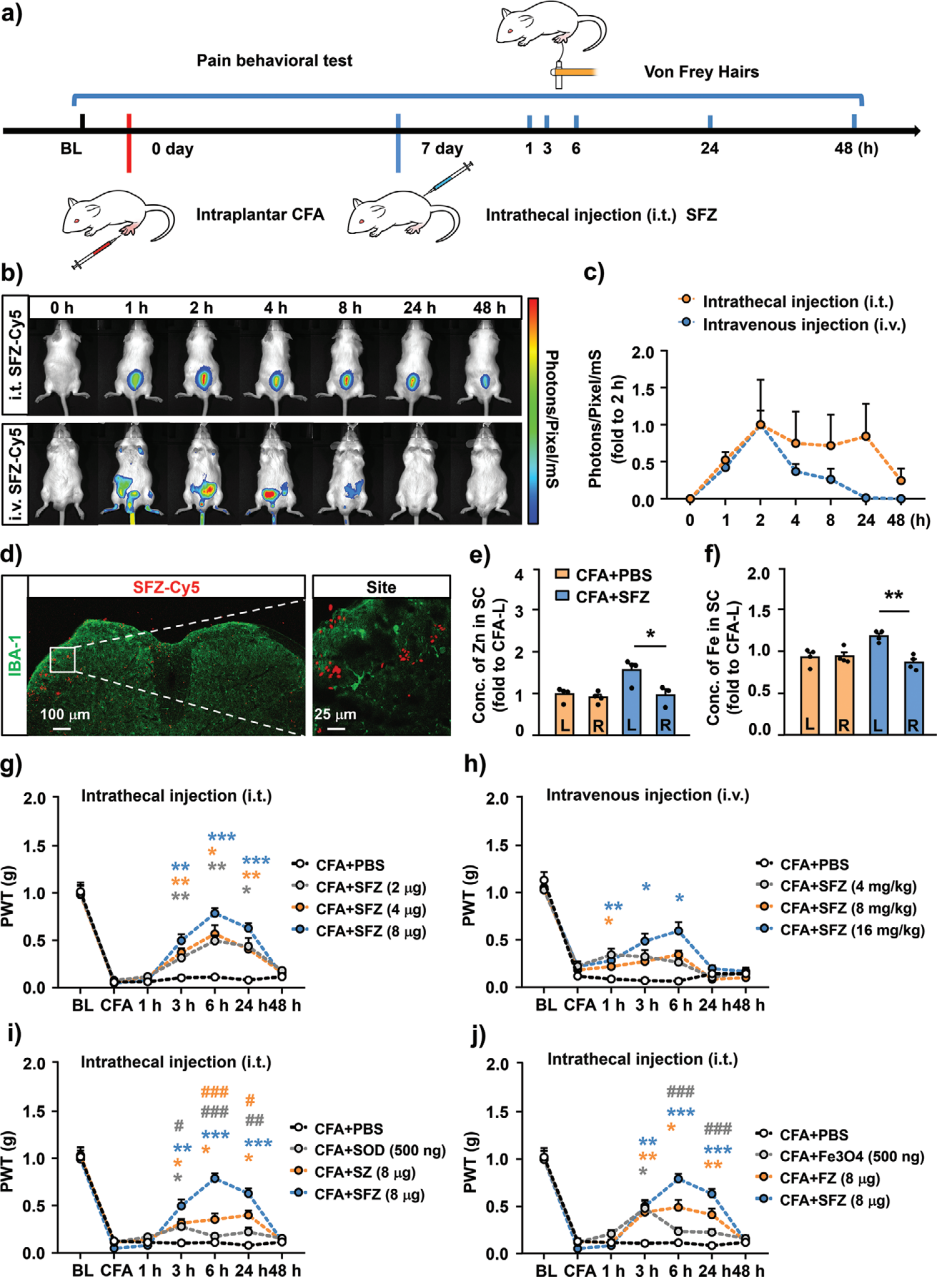
In vivo uptake of SFZ NPs and effects on CFA‐induced inflammatory pain. a) Schematic of a model of CFA‐induced inflammatory pain and pain behavioral tests. b) In vivo fluorescence images of mice taken at different time points injected intrathecally/intravenously with SFZ‐Cy5 NPs. c) Quantification of the distribution of SFZ‐Cy5 NPs assessed as the radiant efficiency of the images. d) Localization of SFZ‐Cy5 NPs in the left side of the spinal cord 6 h after intrathecal injection. Scale bar, 100 µm. The inset to the right shows the accumulation of SFZ‐Cy5 NPs in a surrounding region of spinal microglia cells. Scale bar, 25 µm. e,f) Biodistribution and concentration of Zn/Fe in the spinal cord after intrathecal injection with SFZ NPs. L = Left side of the spinal cord, R = Right side of the spinal cord. *p < 0.05, **p < 0.01, CFA+SFZ versus CFA. Student's *t*‐test (*n* ≥ 3). g,h) Mechanical allodynia of CFA‐induced mice treated with different concentration gradients of SFZ NPs injected g) intrathecally/intravenously (h). Interaction: Intrathecal injection, *F*_(18, 144)_ = 6.213, *p* < 0.0001; intravenous injection, *F*_(18, 162)_ = 3.154, *p* < 0.0001. *p < 0.05, **p < 0.01, ***p < 0.001. CFA+SFZ versus CFA+PBS. Two‐way RM ANOVA followed by Bonferroni's tests (*n* ≥ 6). i,j) Mechanical allodynia of CFA‐induced mice treated with other nanoparticles (SOD, SZ, Fe_3_O_4_, FZ, and SFZ NPs) injected intrathecally. Interaction: i) *F*_(18, 144)_ = 9.655, *p* < 0.0001; j) *F*_(18, 144)_ = 9.020, *p* < 0.0001. *p < 0.05, **p < 0.01, ***p < 0.001. *^#^p* < 0.05, *^##^p* < 0.01, *^###^p* < 0.001. The different‐colored lines indicate different groups. The asterisks (*) of the same color indicated their respective comparison with the CFA+PBS group. The hashtag (#) of the same color indicated their respective comparison with the CFA+SFZ group. Two‐way ANOVA followed by Bonferroni's test (*n* = 7).

The timeline of the in vivo experimental procedure is shown in **Figure** **5**a. We determined the distribution of SFZ NPs in vivo using fluorescence detection to observe SFZ‐Cy5 NPs in mice injected intrathecally and intravenously. A single intrathecal injection of SFZ‐Cy5 NPs maintained a high concentration at the injection site within 24 h and did not decay until 48 h. In contrast, intravenous injection of SFZ‐Cy5 NPs began to decay 4 h after injection and only lasted for 24 h (Figure 5b,c). Furthermore, colocalization with the microglial marker IBA1 showed that intrathecal injection of SFZ‐Cy5 NPs could accumulate around the glial cells in the spinal cord on the inflamed side (Figure 5d). To confirm the successful delivery of SFZ NPs into the L5 spinal cord, Zn and Fe were quantified in the spinal cord using the ICP method after intrathecal injection of SFZ NPs. Quantitative results showed that 6 h after intrathecal injection of SFZ NPs, the content of Zn and Fe in the spinal cord of the SFZ NPs group was higher than those of the phosphate‐buffered saline (PBS) group. Additionally, the content on the left side of the spinal cord (modeling side) was ≈1.5 times that of the right side (Figure 5e,f). These results suggest the successful delivery of SFZ NPs to the L5 spinal cord and their tendency to accumulate in the spinal cord's inflammatory side. This may be attributed to the increased vascular permeability and enhanced endocytosis of glial cells in an inflammatory environment.

### 2.7 Intrathecal Injection of SFZ NPs Significantly Relieved Inflammatory Pain

To assess the in vivo analgesic efficacy of SFZ NPs in mice, we measured mechanical nociceptive hypersensitivity by the withdrawal response on the hind paw foot surface after stimulation with calibrated von Frey filaments.^[^ ^29^ ^]^ We used the premise of pain behavior assessment as the normal motor coordination ability of mice. In other words, mice can respond to noxious stimuli under normal circumstances by withdrawing their paws and raising their grasp.^[^ ^30^ ^]^ Compared with the PBS group, the mice had the same tail‐flick latency, paw withdrawal threshold (PWT), and latency to get off the rotator after intrathecal injection of SFZ NPs (Figure S9, Supporting Information). This demonstrates that the intrathecal injection of NPs did not interfere with the motor coordination ability of the mice. Moreover, on the 3rd day after CFA injection, the mechanical allodynia of mice decreased significantly. Additionally, intrathecal injection of SFZ NPs (2 µg/10 µL, 4 µg/10 µL, and 8 µg/10 µL) resulted in a dose‐dependent reversal of hyperalgesia after 3 h, and the effect was maintained for 24 h. The high‐dose group had the best analgesic effect, maintained until 6 h, consistent with the time SFZ‐Cy5 NPs reside in vivo (Figure 5g,h). Moreover, the analgesic effects of ZIF‐8‐encapsulated nanocomposites, especially co‐encapsulated SOD and Fe_3_O_4_, were better than those of free SOD and Fe_3_O_4_ NPs and equal to that of dexamethasone (positive control) (Figure 5i,j). This validated the cascade catalytic activities of SFZ NPs to relieve the inflammatory reaction for acesodyne. Additionally, SFZ NPs could inhibit the neuropathic pain induced by L5 spinal nerve ligation and spare nerve injury.^[^ ^31^ ^]^ Furthermore, 7 days after nerve injury, intrathecal injection of SFZ NPs for 6 h significantly relieved mechanical hyperalgesia in mice (Figure S10, Supporting Information). These demonstrate the successful delivery of SFZ NPs to the spinal cord after intrathecal injection, resulting in an excellent analgesic effect.

### 2.8 SFZ NPs Inhibits CFA‐Induced Glial Cell Activation and Inflammatory Signaling Pathways in the Spinal Cord Caused by Inflammatory Pain

**Figure 6:**
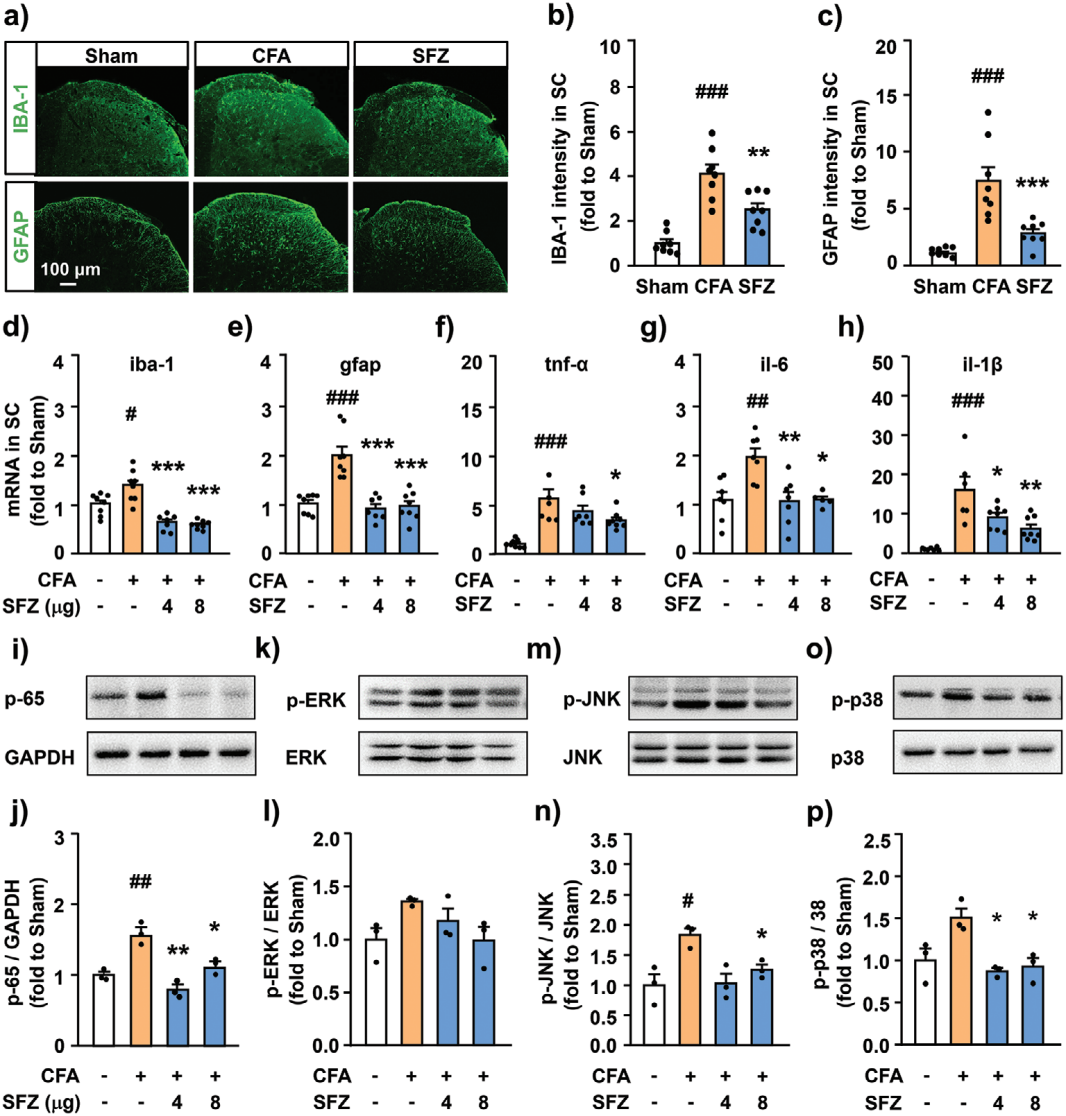
Protection of SFZ NPs against neuroinflammation in the spinal cord. a) Representative images of IBA‐1 and GFAP immunofluorescence in the dorsal horn of the spinal cord with different treatments (*n* = 8, from three mice). b,c) Quantification of fluorescence intensity of b) IBA‐1 and c) GFAP. d,e) The mRNA levels of iba‐1 and gfap in the spinal cord with different treatments. *^#^p* < 0.05, *^###^p* < 0.001, t‐BOOH versus PBS. ***p < 0.001, versus t‐BOOH, one‐way ANOVA, followed by Bonferroni's test (*n* ≥ 7). f–h) The mRNA levels of f) TNF‐*α*, f) IL‐6, and h) IL‐1*β* in the spinal cord with different treatments. *^##^p* < 0.01, *^###^p* < 0.001, t‐BOOH versus PBS. *p < 0.05, **p < 0.01, versus t‐BOOH, one‐way ANOVA, followed by Bonferroni's test (*n* ≥ 6). i–p) Expression and protein quantification of i,j) p‐65, k,l)p‐ERK, m,n) p‐JNK, and o,p) p‐p38 in the spinal cord with different treatment characterized by western blotting. *^#^p* < 0.05, *^##^p* < 0.01, t‐BOOH versus PBS. *p < 0.05, **p < 0.01, versus t‐BOOH, one‐way ANOVA, followed by Bonferroni's test (*n* = 3).

Inflammatory pain causes the activation and proliferation of many glial cells in the spinal cord, resulting in a series of inflammation‐related signaling pathways leading to severe neuroinflammation.^[^ ^32^ ^]^ Among them, astrocytes and microglia are the primary inflammatory cells in the spinal cord, and their activation reflects the inflammatory state in the spinal cord. Therefore, we determined the expression levels of GFAP and IBA‐1 in the L5 spinal cord using immunohistochemical staining and quantitative PCR, respectively. Confocal results showed that the fluorescence intensities of GFAP and IBA‐1 in the spinal dorsal horn of CFA mice were significantly increased compared with those of the sham group, illustrating the induced astrocyte and microglia activation by CFA. In this case, SFZ NPs were intrathecally injected into CFA mice. After 6 h, the number of GFAP and IBA‐1 positive cells was significantly reduced (**Figure** **6**a–c), consistent with the PCR results, where the GFAP and IBA‐1 mRNA expression levels in CFA‐induced mice could be significantly attenuated after intrathecal injection of SFZ NPs (Figure 6d,e).

In addition, we examined the expression levels of TNF‐*α*, IL‐6, and IL‐1*β* in the spinal cord. Figure 6f–h shows the enhanced secretions of TNF‐*α*, IL‐6, and IL‐1*β* in the spinal cord induced by the CFA model compared with those in the sham group. However, after treatment with SFZ NPs for 6 h, the levels of pro‐inflammatory cytokines showed a dose‐dependent decrease. Furthermore, we examined the activation of inflammation‐related signaling pathways in the spinal cord using immunoblotting. Figure 6i–p shows the significantly increased expression levels of p‐65, p‐ERK, p‐JNK, and p‐p38 in the spinal cord of CFA mice, reversed after treatment with SFZ NPs. All the above results indicated that SFZ NPs could effectively inhibit the activation of astrocytes and microglia, reverse the MAPK signaling pathway's phosphorylation process, and alleviate neuroinflammatory damage/pain.

Finally, the biosafety problem of new NPs cannot be ignored for its future biomedical applications. Therefore, we examined the in vivo toxicity of intravenously injected SFZ NPs in healthy mice using hematological and pathological techniques. Results from serum biochemical indexes and H&E staining in mice showed that the major organs, such as the heart, liver, spleen, lung, kidney, and brain, did not show obvious inflammation or other pathological changes after 28 days of treatment with SFZ NPs (Figure S11, Supporting Information). Additionally, intrathecal injection of SFZ NPs did not cause any adverse effects on the nervous system behavior of mice or histological changes of the spinal cord. These indicate the good biocompatibility and safety of SFZ NPs, which would be a potential novel analgesic agent for future applications.

## 3 Conclusion

We prepared a safe cascade nanozyme by homogeneously incorporating SOD and Fe_3_O_4_ NPs into ZIF‐8 NPs through a simple co‐assembly strategy. Due to the degradation‐induced exposures of active sites in the weakly acidic inflammatory environment and cascade SOD‐ and CAT‐like enzyme activities, this SFZ nanoenzyme could maintain spinal glial cells in a quiescent state by reducing oxidative stress and regulating the pro‐inflammatory microenvironment, thereby treating inflammatory pain. Furthermore, in vitro and in vivo investigations confirmed that this process involved the accumulation of SFZ NPs around the activated glia cells in the spinal cord following intrathecal injection, the conversion of ROS into O_2_, and the reduction of neuroinflammatory damage by modulating MAPK/p‐65 signaling pathway. Therefore, this study provides a novel antioxidant and anti‐inflammatory NP and elucidates the molecular mechanisms of anti‐inflammatory pain and the inhibition of glial cell activation, potentially providing new strategies for treating neuroinflammatory diseases.

## 4 Experimental Section

### The Synthesis of Fe_3_O_4_ NPs

First, two solutions (A and B) were prepared. Solution A was a 4 mg mL^−1^ poly acrylic acid (PAA) solution prepared by dissolving 0.08 g PAA (Mw = 1800) in 20 mL deionized water. Solution B was comprised of 0.2 mL FeCl_3_ (500 mm) and 0.2 mL FeSO_4_·7H_2_O (250 mm) solutions and needed to be freshly prepared for use. Before adding solution B to A, the O_2_ in solution A was removed by passing N_2_ for ≈50 min. Furthermore, solution A required heating reflux at 100 °C, and solution B was added quickly. After that, 6 mL ammonia was added to the reaction, which was maintained at 100 °C for 1 h. Next, it was wrapped up and cooled to room temperature (RT), and dialysis was performed for 3 days. Finally, the mixture was freeze‐dried to obtain Fe_3_O_4_ NPs.

### The Synthesis of ZIF‐8/SZ/FZ/SFZ/SFZ‐Cy5 NPs

In the first step, 43.9 mg zinc acetate dihydrate was dissolved in 10 mL deionized water and stirred for 15 min. Next, Fe_3_O_4_ solution was prepared by dissolving 2.5 mg Fe_3_O_4_ in 2.5 mL H_2_O. In the second step, the Fe_3_O_4_ solution was added to the system and stirred for 15 min. In the third step, 10 mL 2‐methylimidazole (0.12 g mL^−1^) was added rapidly. Afterward, the system was continuously stirred for 10 h at 30 °C to get the solution. FZ NPs precipitate was obtained after centrifugation, which was washed thrice using ethanol and H_2_O, and freeze‐dried to obtain a dried FZ NPs sample. Next, ZIF‐8 NPs, SZ NPs, and SFZ NPs were obtained after adding alternative Fe_3_O_4_ solutions to H_2_O, 2 mL H_2_O, SOD solution (2.5 mg SOD dissolved into 0.5 mL H_2_O), Fe_3_O_4_ (2.5 mg Fe_3_O_4_ dissolve into 2 mL H_2_O), and SOD solutions (2.5 mg SOD dissolve into 0.5 mL H_2_O), respectively. Additionally, SFZ‐Cy5 NPs was obtained by adding another 1 mg of Cy5 to the SFZ NPs solution.

### Material Characterizations

TEM was carried out on FEI Talos F200S, and high‐resolution TEM, STEM, and EDX mappings of the catalysts were performed on a JEM‐2100 electron microscope (JEOL, Japan) with an acceleration voltage of 200 kV. Additionally, field emission scanning electron microscopy observations were performed using a Regulus 8230 microscope (Hitachi, Japan). Moreover, XRD analysis was acquired on a D/MAX 2550 VB/PC diffractometer (Rigaku, Japan), and XPS was performed on an RBD‐upgraded PHIe5000C ESCA system (PerkinElmer, USA). Furthermore, FTIR was recorded in the range of 400–4000 cm^−1^ on a VERTEX 70 spectrometer (Bruker, Germany). Furthermore, TGA was performed using a DISCOVERY TGA 550 system (TA Instruments, USA) over 30–900 °C under an air atmosphere. Finally, Raman spectra were captured on a Renishaw confocal microscope Raman spectrometer (Renishaw, UK). Elemental contents were determined using inductively coupled plasma atomic emission spectroscopy (Prodigy ICP‐AES).

### Detection of Oxygen Generation

ZIF‐8 NPs, Fe_3_O_4_ NPs, FZ NPs, and SFZ NPs were separately exposed to H_2_O_2_ (1 mm), where the oxygen amounts were in real‐time monitored using a dissolved oxygen meter (JPBJ‐609L). The generation of O_2_ was measured at different time points.

### Cell Culture

BV2 microglial cells were purchased from the Chinese Academy of Sciences. BV2 cells were cultured in a complete medium comprising 10% fetal bovine serum (Gibco), high glucose Dulbecco's Modified Eagle's Medium (DMEM, Gibco), and 1% penicillin–streptomycin. Additionally, primary microglial cells were cultured as described previously. Furthermore, the cerebral hemispheres of day 1–3 neonatal mice were isolated, minced in pre‐chilled buffer, and filtered through a 100 µm nylon mesh. Next, the filtered cell suspension was inoculated into 75 cm^2^ culture flasks and cultured in the complete medium described above. After 2 weeks, microglia were isolated by shaking flasks at 220 rpm and 37 °C for 4 h. Finally, microglia were seeded into the desired vessel. DMEM was replaced with reduced serum medium (Opti‐MEM, Invitrogen) before drug treatment.

### In Vitro Cytotoxicity Assay

100 µL of BV2 cell suspension was cultured in a 96‐well plate at 5000 cells/well for 12 h. After the cells adhered, they were used for cytotoxicity assay. Gradient concentrations (2, 4, 8, 16, 32, and 64 µg mL^−1^) of ZIF‐8 NPs, SOD, Fe_3_O_4_ NPs, SOD@ZIF‐8 NPs, Fe_3_O_4_@ZIF‐8 NPs, and SOD&Fe_3_O_4_@ZIF‐8 NPs were added to the 96‐well plate and incubated. After 24 h, the cells were washed twice with PBS, and 10 µL of CCK‐8 solution was added, followed by incubation for 2 h. Finally, CCK‐8 absorbance was measured at 450 nm using a microplate reader.

### Intracellular Uptake of SFZ NPs

BV2 cells were seeded into 6‐well plates. After cell adhesion, the cells were incubated with different concentrations (2, 4, 8, and 16 µg mL^−1^) of SFZ‐Cy5 NPs for 6 h and 16 µg mL^−1^ of SFZ‐Cy5 NPs for different times (1, 2, 4, and 6 h). After removing the medium, the cells were washed thrice with cold PBS to remove extracellular particles, and 0.25% trypsin was added to suspend the cells. Next, the cell suspension was collected and washed thrice with PBS to remove residual medium and trypsin. After resuspending the cells in PBS, the cell suspension was analyzed by flow cytometry for Cy5 fluorescence intensity, enabling the determination of SFZ NPs uptake by BV2 cells.

### Real‐Time Living Cell Imaging

Based on the red fluorescence intensity of Cy5 in SFZ‐Cy5 NPs, the real‐time localization of SFZ‐Cy5 NPs in BV2 cells was observed by live cell imaging. Briefly, the cells were seeded in live cell culture dishes designed for confocal microscopy. Next, 16 µg mL^−1^ of SFZ‐Cy5 NPs was added to 1 mL opti‐MEM after the cells adhered. Subsequently, 1, 2, 4, and 6 h after adding NPs, images were taken with a confocal microscope. Simultaneously, the lysosomes of BV2 cells were stained with a green fluorescent lysosome tracker.

### Internalization of SFZ NPs in BV2 Cells by TEM

BV2 cells were seeded into 10 cm dishes. After cell adhesion, SFZ NPs (16 µg mL^−1^) were added and incubated for 6 h. After rinsing thrice with cold PBS, the adherent cells were gently scraped from the dish using a self‐provided rubber spatula. Next, the collected cell suspension was centrifuged, the supernatant was discarded, and 2.5% glutaraldehyde was added for fixation. Finally, the samples were embedded, sectioned, and observed under TEM. Kindly refer to the manual for specific operations.

### Evaluation of Intracellular ROS

BV2 cells were seeded in 24‐well plates (NEST Biotechnology, China). After cell adhesion, the different nanoparticles group was incubated for 4 h, and 40 µm t‐BOOH was added to both the model group and the nanoparticle group and incubated for 1 h to induce oxidative stress. At the same time, a blank control group was set without t‐BOOH and NPs. Finally, all groups were simultaneously stained with a 10 µm H2DCFDA probe for 30 min, and the cell suspension was collected according to the above method. The fluorescence intensity of intracellular H2DCFDA was analyzed using flow cytometry. The intensity of ROS was analyzed according to the fluorescence intensity. Likewise, BV2 cells were seeded in confocal dishes. Added nanoparticles and drugs according to the above methods and steps. Finally, the fluorescence intensity of the H2DCFDA probe was observed by a confocal microscope.

### Evaluation of Intracellular O_2_ Level

The culture and administration of BV2 cells were consistent with the above. Finally, all groups were simultaneously stained with a 5 µm Ru(dpp)_3_]Cl_2_ probe for 30 min, and the cell suspension was collected according to the above method. The fluorescence intensity of intracellular Ru(dpp)_3_]Cl_2_ was analyzed using flow cytometry and a confocal microscope.

### Animals and Inflammatory Pain Model

Adult male ICR mice weighing 20–28 g were provided (Experimental Animal Center of Nantong University, Nantong, China). Animals were kept under a humidity‐controlled environment (26 °C, 12 h light/dark cycle) with free‐feeding. All animal procedures were performed according to the protocols of the National Institutes of Health Guidelines for the care and use of laboratory animals. The statement that ethical approval from the Experimental Animal Center of Nantong University was obtained, the accreditation number is S20221118‐017. After the mice were briefly anesthetized with isoflurane, inflammatory pain was induced by intraplantar injection of 20 µL CFA (Sigma–Aldrich) into the left hind paw.

### Behavioral Tests

Regarding the von Frey test, the animals were placed in boxes on an elevated metal mesh floor daily for at least 2 days before baseline testing, and 30 min was allowed for habituation before the examination. Next, the plantar surface of the left hind paw was stimulated with a series of von Frey hairs with logarithmically incrementing stiffness (0.02–2.56 g, Stoelting, Wood Dale, IL). Afterward, the 50% PWT was determined using Dixon's up‐down method. The behavior graph ordinate was the PWT. All the behavioral experiments were done by individuals blinded to the treatment of the mice.

### Biodistribution of Nanoparticles in the Spinal Cord

After brief gas anesthesia, the mice were bent over and placed in an in vivo imaging system (Tanon ABL X6, China). Images were collected at 1, 2, 4, 8, 24, and 48 h after intrathecal administration of SFZ NPs (10 µg/10 µL). Fluorescence images were acquired using Tanon Live Animal Analysis System version 6.50 (Tanon, CN).

### Western Blotting

After brief anesthesia with isoflurane, the animals were perfused with PBS, and L 4–6 spinal cords were dissected. Cells or spinal cords were homogenized by adding an appropriate amount of lysis buffer (containing protease and phosphatase inhibitors). Protein supernatants were assayed for protein concentration using the BCA protein assay (Pierce, Rockford, IL). 30 µg of total protein was loaded per channel and separated on 10% or 12% SDS‐PAGE gels. After transfer and blocking with 5% BSA, blots were incubated overnight at 4 °C with the following primary antibodies: anti‐p‐ERK (rabbit, 1:1000, CST), anti‐p‐p38 (rabbit, 1:1000, CST), anti‐p‐JNK (rabbit, 1:1000, CST), and anti‐p‐65 (rabbit, 1:1000, CST). These blots were separately incubated with ERK (rabbit, 1:1000, CST), p‐38 (rabbit, 1:1000, CST), JNK (rabbit, 1:1000, CST), and GAPDH antibody (mouse, 1:20 000, Sigma) as a loading control. Then the membranes were incubated with secondary antibodies (Dylight 800, Donkey Anti‐Rabbit or Anti‐Mouse IgG, 1:10 000, Millipore). The bands were detected using the Odyssey System (Li‐COR, NE, USA). Finally, protein quantification using Image J (NIH, MD, USA).

### Real‐Time Quantitative PCR

Total RNA from the spinal cord and cultured cells was extracted for mRNA detection using Trizol reagent (Invitrogen). Next, according to Takara's protocol, 1 µg of total RNA was reverse transcribed to cDNA using oligo (dT) primers. Additionally, qPCR analysis was performed using SYBR Green I Dye Detection on a real‐time detection system (Rotor‐Gene 6000, Hamburg, Germany). Primer sequences for TNF‐*α*, IL‐1*β*, IL‐6, IBa‐1, Gfap, and Gapdh are as follows: TNF‐*α* forward, 5″‐GTT CTA TGG CCC AGA CCC TCA C‐3″, and reverse, 5″‐GGC ACC ACT AGT TGG TTG TCT TTG‐3″; IL‐1*β* forward, 5″‐TCC AGG ATG AGG ACA TGA GCA C‐3″, and reverse, 5′‐ GAA CGT CAC ACA CCA GCA GGT TA ‐3′; IL‐6 forward, 5″‐TAG TGG ATG CTA CCA AAC TGG A‐3″, reverse, 5″‐TGT GAC TCC AGC TTA TCT CTT G G‐3″; IBa‐1 forward, 5″‐ATG AGC CAA AGC AGG GAT T‐3″, reverse, 5″‐CTT CAA GTT TGG ACG GCA G‐3″; Gfap forward, 5″‐GGC GAA GAA AAC CGC ATC AC‐3″, reverse, 5″‐CTC CAC CGT CTT TAC CAC GA‐3″; Gapdh forward, 5″‐AAA TGG TGA AGG TCG GTG TGA AC‐3″, and reverse, 5″‐CAA CAA TCT CCA CTT TGC CAC TG‐3″. The PCR amplifications were performed at 95 °C for 30 s, followed by 40 cycles of thermal cycling at 95 °C for 5 s and 60 °C for 45 s. Gapdh was used as an endogenous control to normalize differences for mRNA. Quantification was performed by normalizing Ct (cycle threshold) values with Gapdh Ct (mRNA) and analyzed with the 2^−ΔΔCT^ method.

### Immunohistochemistry and Immunocytochemistry

The animals were anesthetized by isoflurane followed by perfusion via the ascending aorta with saline and 4% PFA. After perfusion, the associated tissue was removed and fixed in 4% PFA overnight at 4 °C. Finally, the tissue was dehydrated in a 20% and 30% sucrose solution gradient. Spinal cords (30 µm) were cut in 2% goat serum for 1 h at RT and then incubated overnight at 4 °C with the primary antibodies. The antibodies information was listed as follows: IBA‐1 (goat, 1:3000, Abcam), GFAP (mouse, 1:5000, Millipore). FITC‐or Cy3‐conjugated secondary antibody (1:400, Jackson ImmunoResearch, West Grove, PA). After rinsing with PBS, incubated by a mixture of FITC‐ and Cy3‐conjugated secondary antibodies for 1 h at room temperature and then examined with a Leica fluorescence microscope. For immunocytochemistry, cultured cells were fixed with 4% PFA for 20 min, and processed for immunofluorescence with anti‐IBA‐1 and anti‐GFAP primary antibodies as shown earlier. After immunostaining, DAPI (0.1 µg mL^−1^, Sigma) was added for 5 min at RT to stain all the cell nuclei.

### In Vivo Biosafety Assessment

SFZ NPs (20 mg kg^−1^) were intravenously injected into mice (*n* = 3). 28 days after injection, mice were sacrificed and blood was collected to obtain routine data. At the same time, major organs, including the heart, liver, spleen, lung, kidney, and brain, were removed and processed for H&E staining.

### Statistical Analysis

All data were analyzed by researchers blinded to the reagents used. All data were analyzed by GraphPad Prism (version 8.02) and presented as mean ± SEM. *p* < 0.05 was considered statistically significant. Behavioral data were analyzed using two‐way ANOVA. Other experimental data were compared using one‐way ANOVA. Student's *t*‐test was used to analyze data if only two groups were applied.

## Conflict of Interest

The authors declare no conflict of interest.

## Supporting information

Supporting Information

## Data Availability

The data that support the findings of this study are available from the corresponding author upon reasonable request.
