# Supplementary material for: Antioxidant Cascade Nanoenzyme Antagonize Inflammatory Pain by Modulating MAPK/p‐65 Signaling Pathway
Source: Adv Sci (Weinh). 2023 Feb 17;10(12):2206934. doi: 10.1002/advs.202206934 (PMC10131840; doi:10.1002/advs.202206934)
Supplement: Supplementary file 1 — Supporting Information [file ADVS-10-2206934-s001.pdf]

ADVANCED SCIENCE
Open Access

Supporting Information

for *Adv. Sci.*, DOI 10.1002/advs.202206934

Antioxidant Cascade Nanoenzyme Antagonize Inflammatory Pain by Modulating MAPK/p-65 Signaling Pathway

Yuejuan Ling, Dekang Nie, Yue Huang, Mengyuan Deng, Qianqian Liu, Jinlong Shi, Siguang Ouyang, Yu Yang, Song Deng, Zhichao Lu, Junling Yang, Yi Wang, Rongqin Huang* and Wei Shi**

Supporting Information

Antioxidant Cascade Nanoenzyme Antagonize Inflammatory Pain by Modulating MAPK/p-65 Signaling Pathway

Yuejuan Ling, Dekang Nie, Yue Huang, Mengyuan Deng, Qianqian Liu, Jinlong Shi, Siguang Ouyang, Yu Yang, Song Deng, Zhichao Lu, Junling Yang, Yi Wang, Rongqin Huang,* and Wei Shi**

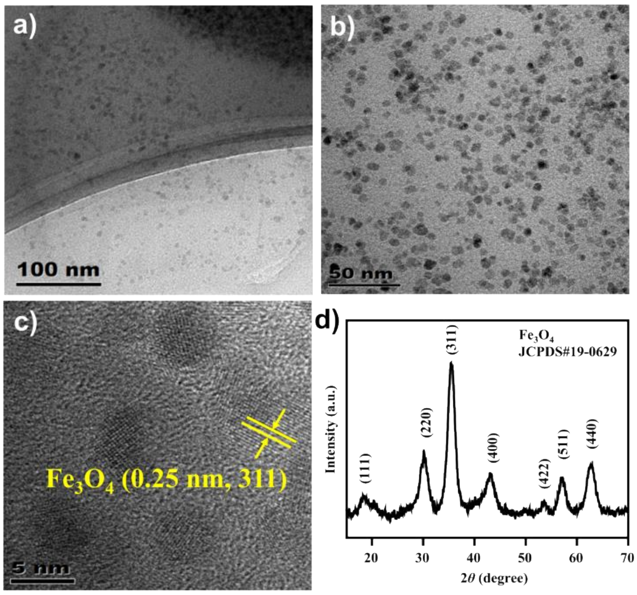

Figure S1. a, b) TEM images with different magnifications, c) HRTEM image and d) XRD pattern of the as-synthesized Fe_3O_4 NPs. The obvious lattice fringes and distinct diffractions revealed the crystalline Fe_3O_4 NPs, while the wide XRD peaks also implied the small particle sizes.

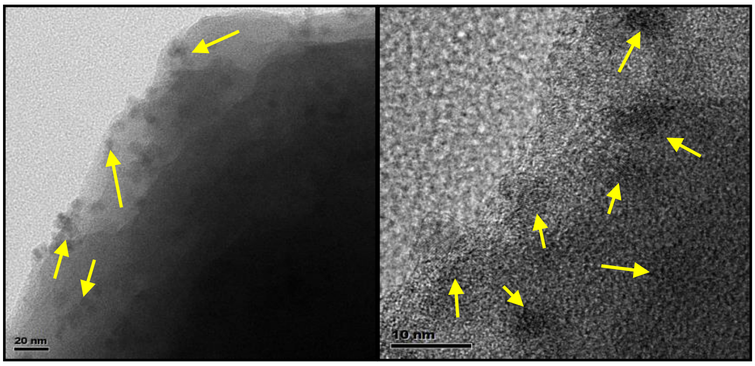

Figure S2. HRTEM images of SFZ NPs. The arrows showed the uniformed incorporations of crystalline Fe_3O_4 NPs into SFZ NPs.

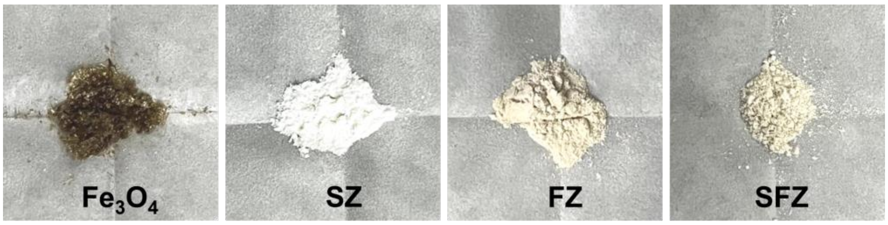

Figure S3. Photos of different nano-preparations.

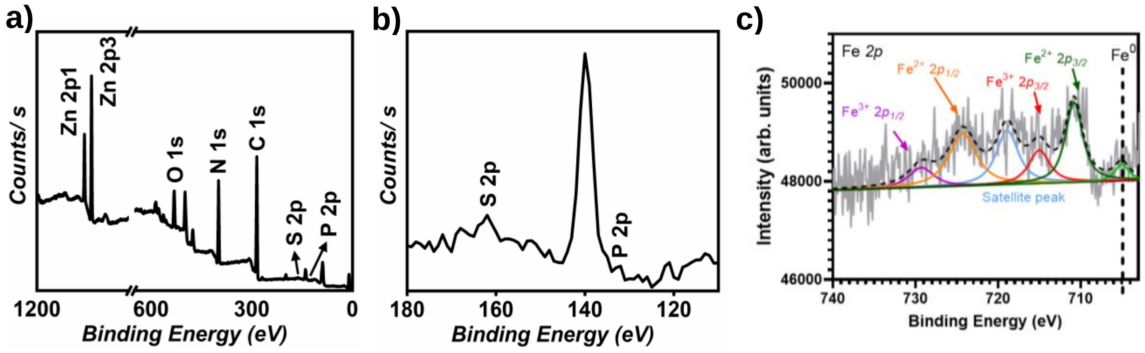

Figure S4. a) XPS survey and b, c) the corresponding high-resolution S 2p and Fe 2P spectra of SFZ NPs.

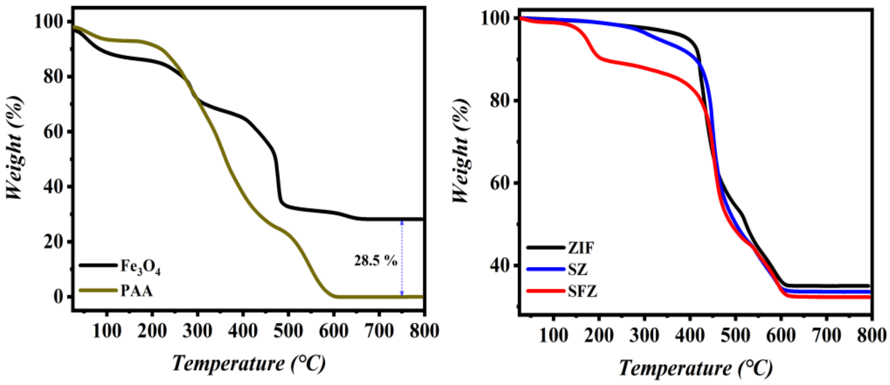

Figure S5. TGA curves in O_2 atmosphere of different nano-preparations. The obvious weight loss for Fe_3O_4 suggested some residual organic groups that were stably anchored on the Fe_3O_4 NPs and can't be removed via washing. This facilitated the water dispersion of Fe_3O_4 NPs and its incorporation into ZIF-8 NPs.

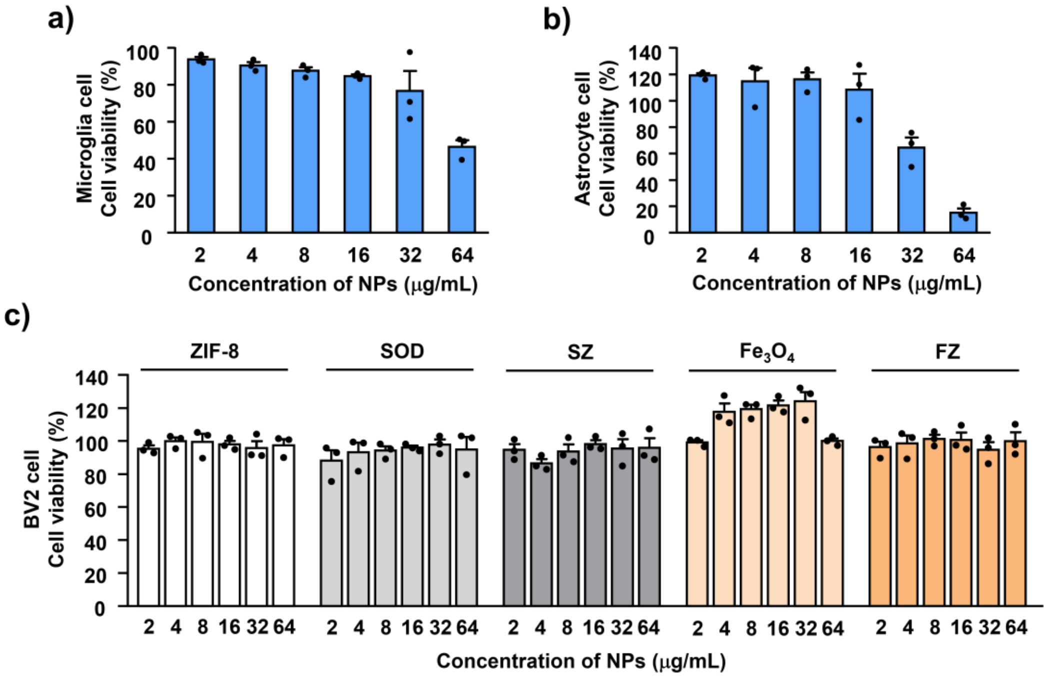

Figure S6. The cell viability of SFZ NPs in primary microglia cells and primary astrocyte cells. a) The cell viability of primary microglia cells with SFZ NPs of different concentration for 24 h (n = 3). b) The cell viability of primary astrocyte cells with SFZ NPs of different concentration for 24 h (n = 3). c) The cell viability of BV2 cells with ZIF-8, SOD, SZ, Fe_3O_4 and FZ NPs of different concentration for 24 h (n = 3).

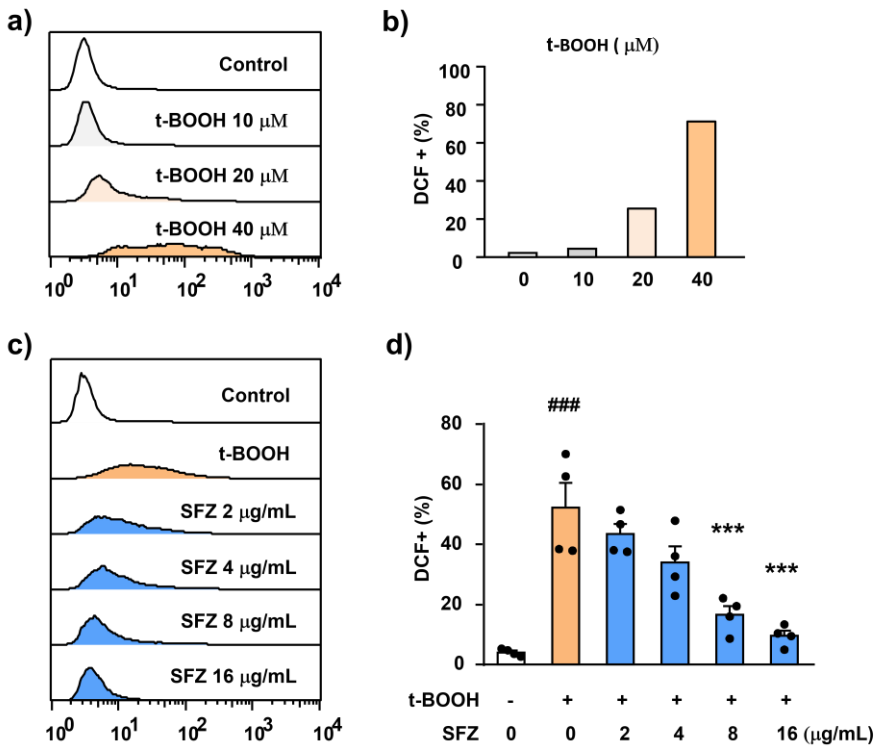

Figure S7. Protection of SFZ NPs against oxidative stress in BV2 cells. a) Representative diagram of the ability of different concentrations of t-BOOH to induce ROS production in BV2 cells. b) quantitative analysis of DCF^+ by flow cytometry. c) Representative graph of SFZ NPs at different concentrations to scavenge ROS induced by t-BOOH in BV2 cells, d) quantitative analysis of DCF^+ by flow cytometry. $^{###}$*P* < 0.001, t-BOOH *vs.* PBS. ****P* < 0.001, *vs.* t-BOOH, one-way ANOVA, followed by Bonferroni's test (n = 4).

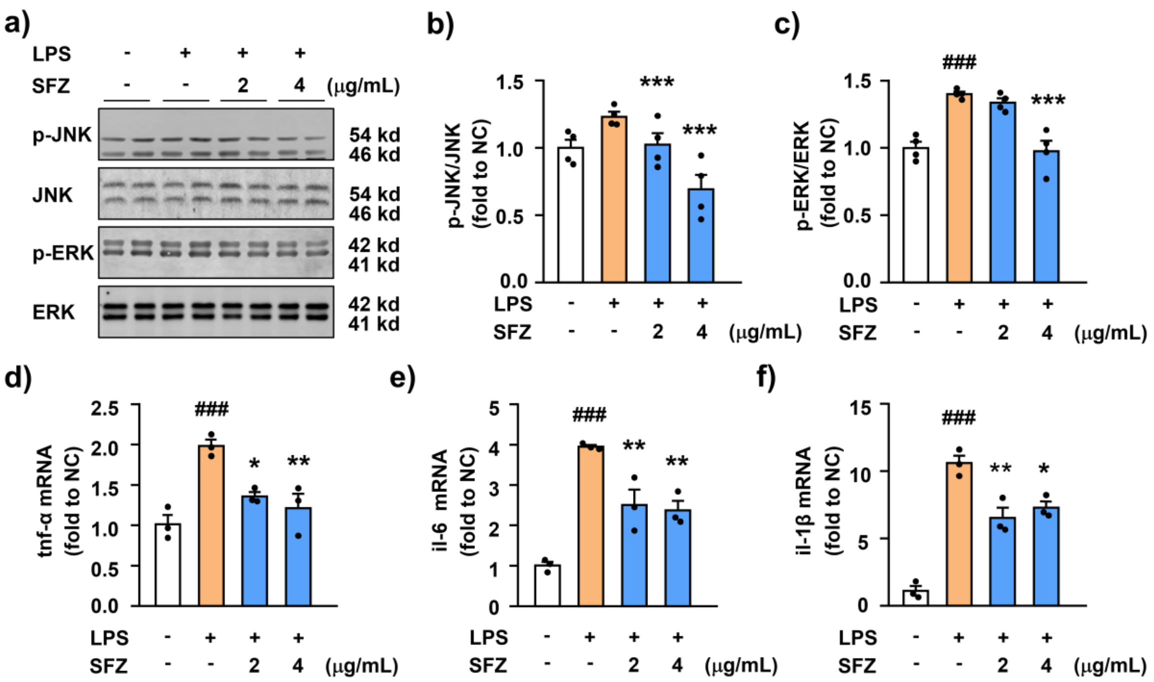

Figure S8. Protection of SFZ nanoparticles against neuroinflammation in primary astrocyte cells. a-c) Expression and protein quantification of p-JNK (a, b) and p-ERK (a, c) in primary astrocyte cells with different treatment characterized by western blotting. $^{###}P < 0.001$, t-BOOH *vs.* PBS. $^{***}P < 0.001$, *vs.* t-BOOH, one-way ANOVA, followed by Bonferroni's test (n = 4). d-f) The mRNA levels of TNF-α (d), IL-6 (e) and IL-1β (f) in primary astrocyte cells with different treatment. $^{###}P < 0.001$, t-BOOH *vs.* PBS. $^{*}P < 0.05$, $^{**}P < 0.01$, *vs.* t-BOOH, one-way ANOVA, followed by Bonferroni's test (n = 4).

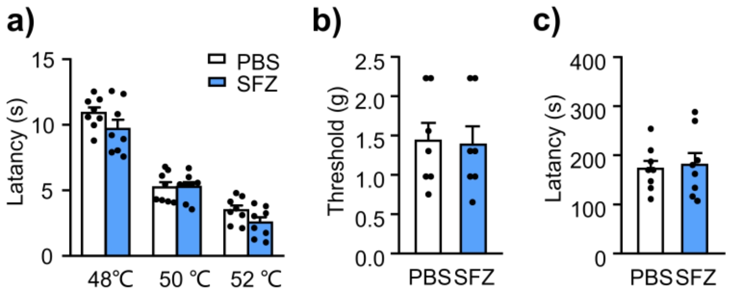

Figure S9. Evaluation of neurotoxicity and motor function in mice after intrathecal injection of SFZ NPs. a-c) Naive mice that received an intrathecal injection of SFZ NPs exhibited a) normal thermal sensitivity (tail-flick test), b) mechanical sensitivity (von Frey test), and c) motor coordination (rotarod test) (n = 8).

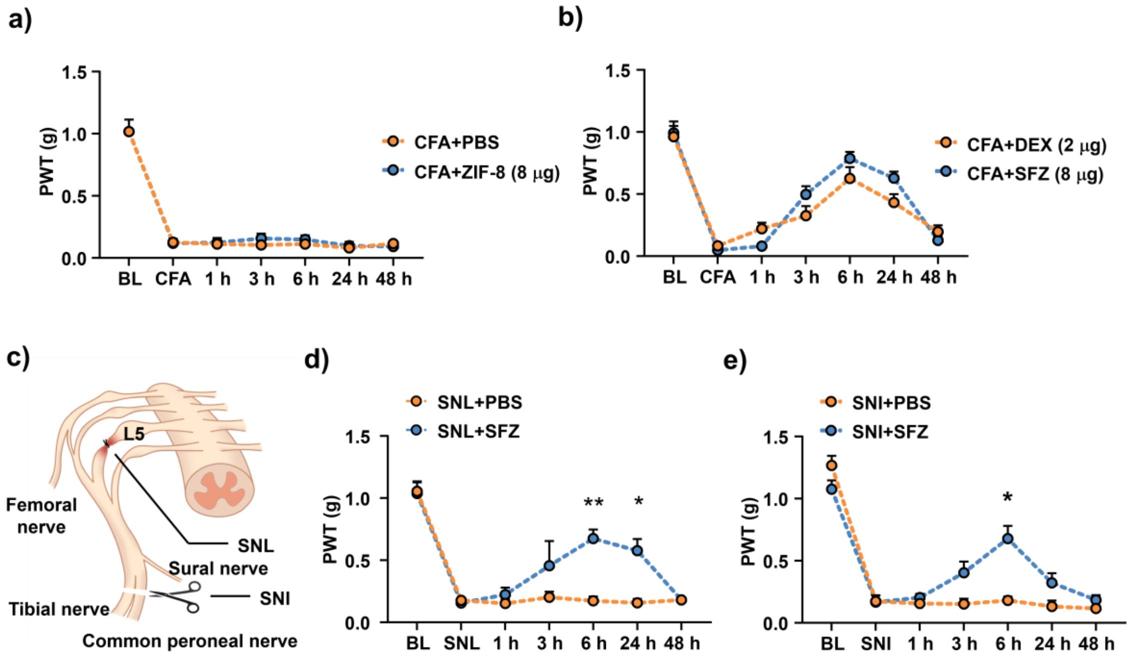

Figure S10. Effects of nanoparticles on CFA-induced inflammatory pain and SNL/SNI-induced neuropathic pain. a, b) Mechanical allodynia of CFA-induced mice treated with nanoparticles (ZIF-8, DEX and SFZ) injected intrathecally. c) Schematic of model of SNL/SNI-induced neuropathic pain. d, e) Mechanical allodynia of SNL/SNI-induced mice treated with SFZ nanoparticles injected intrathecally. Interaction: Figure S10d, $F_{(6, 60)} = 4.468$, P = 0.0008; Figure S10e, $F_{(6, 60)} = 6.686$, P < 0.0001. *$P < 0.05$, **$P < 0.01$. SNL/SNI+SFZ *vs.* SNL/SNI+PBS. The different-colored lines indicate different groups. The asterisks (*) of the same color indicated their respective comparison with the CFA+PBS group. The hashtag (#) of the same color indicated their respective comparison with the CFA+SFZ group. Two-way ANOVA followed by Bonferroni's test (n = 6).

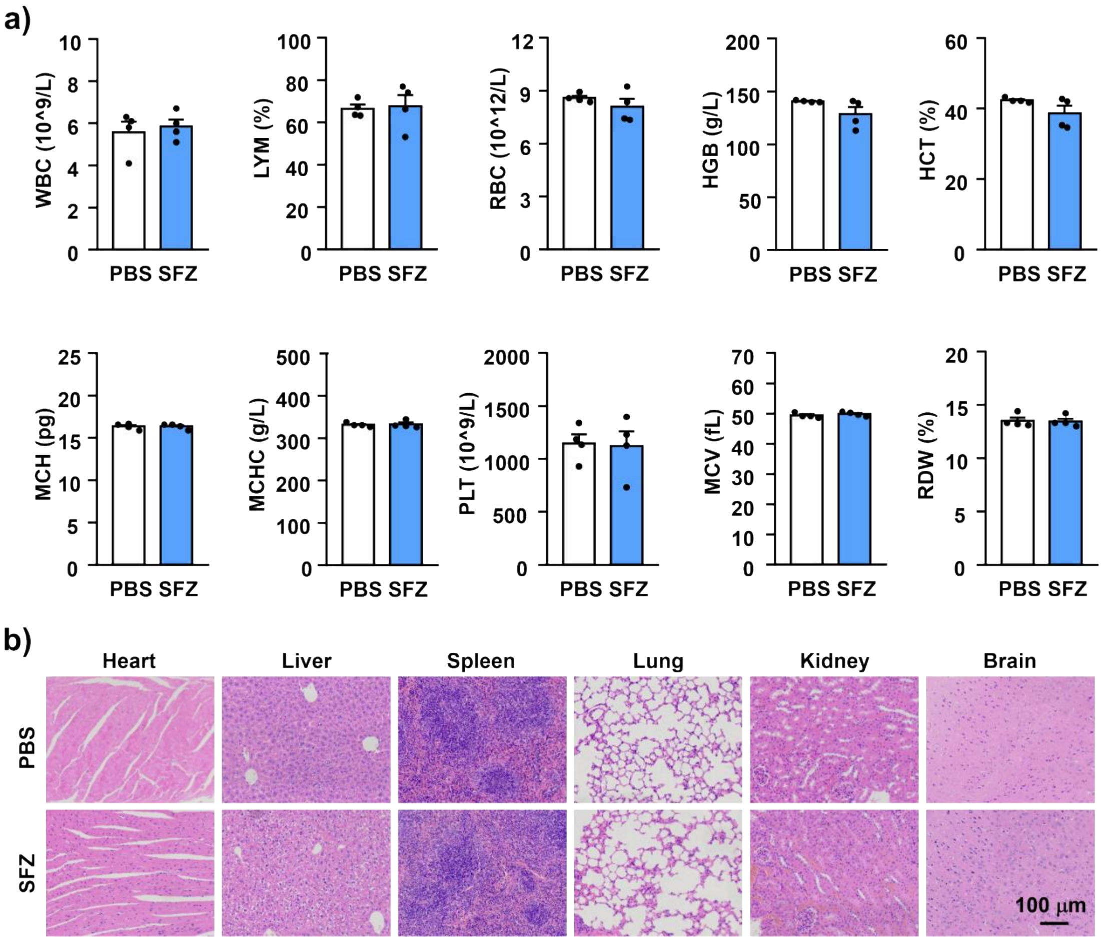

Figure S11. a) Safety evaluation in mice after intravenous injection of SFZ NPs. b) Paraffin-embedded tissue sections were stained with H&E for light microscopy assessment. Scale bar, 100 μm, (n = 3).
